# Morphological Features of PUR-Wood Particle Composite Foams

**DOI:** 10.3390/ma15196741

**Published:** 2022-09-28

**Authors:** Radosław Mirski, Joanna Walkiewicz, Dorota Dukarska, Adam Derkowski

**Affiliations:** Department of Mechanical Wood Technology, Faculty of Forestry and Wood Technology, Poznań University of Life Sciences, 60-627 Poznań, Poland

**Keywords:** polyurethane foams, wood particle, morphological features, structure, X-ray computed tomography, SEM

## Abstract

The aim of this study was to apply waste wood particles (WP) with different sizes from primary wood processing as a filler for open-cell PUR foams. For this purpose, various wood particle sizes were added as a filler for polyurethane foams (PUR). The effects of the addition of of 0.05–<0.125 mm, 0.125–<0.315 mm, 0.315–1.25 mm, and >1.25–2.0 of WP to the polyurethane matrix on the density, the kinetics of PUR foaming, the cell structure, and the morphology were investigated. Scanning electron microscope (SEM) and X-ray computer tomography were used. Based on the results, it was found that the addition of WP in the amount of 10% leads to an increase in density with an increase in particle size. The research shows that the morphology of the PUR-WP foam is influenced by its particle size. The difference in the number and size of cells in PUR-WP composites depends on the wood particle size. The addition of dust causes the formation of cells of much smaller sizes; confirmed by SEM images. Moreover, computer tomography clearly demonstrates that the WP are well-dispersed within the foams’ structures.

## 1. Introduction

Polyurethane foams (PUR) are the product of the polyaddition reaction between polyols and isocyanates with other additives [1]. Because of their broad range of properties, PUR are commonly used in various industry branches, e.g., thermal insulating layers, furniture, and automotive seats [2,3]. There are two main types of foam: rigid foam and flexible polyurethane foam [3]. Nowadays, PUR are the subject of much scientific research. On the one hand, research is underway on the use of new natural substrate sources for their production [4,5]. On the other hand, investigations are conducted on the use of additives and fillers that affect the PUR properties [6,7,8,9].

The literature data clearly indicate the interest of biodegradable components in the PUR foams. Research on the possibility of using natural fillers were conducted. As examples, fillers such as lignin, microcellulose, hemp, wood particles, wood fibers, and starch were investigated [7,10,11,12,13]. The addition of various types of fillers may affect the foam forming process as well as the properties of the finished product. Shan et al., [14] showed that the addition of fillers leads to the increase of the cell density of the foams. Research conducted by De Luca Bossa [15] showed the ability of walnut shell and OH groups of cellulose to react with an isocyanate precursor, which affects the thermal properties of the material. The improvement in thermal stability and the mechanical properties were observed. Moreover, in the case of walnut shell, this causes an increase in stiffness. Chris-Okafor et al., [16] investigated the effect of corn cob and coconut husk on the properties of flexible polyurethane foams (FPUF). It was observed that a density and compression test result increased with increasing filler concentration (5%, 10%, 15%, 20%, 25%). Kenaf fiber loadings (30% weight percent) exhibited the best tensile strength in comparison with other amounts of fillers (20%, 40%, 50%) [17]. As the amount of rapeseed cakes increased, the compressive strength of foams also increased [8]. Zieleniewska et al. [18] investigated rigid polyurethane foam composites with egg shell waste and research has shown the improvement in their mechanical properties and thermal stability. A better thermal stability of PUR foams with filler was also observed by Strąkowska et al. [19], where sugar beet pulp (BP) impregnated with aminopropylisobutyl polyhedral oligomeric silsesquioxanes (APIB-POSS) was applied. Furthermore, the addition of hazelnut shells and walnut shells resulted in an improved thermal stability of PUR foams [20]. Other interesting properties such as the insulation and sound absorption properties can be enhanced as a result of fillers additions [21,22,23].

It is also worth paying attention to how the structure of PUR foam influences its properties. According to the literature data, the use of lignocellulose filler results in weaker cell structures with a large number of open cells, which significantly affects the final properties of polyurethane composites [24]. Sung and Kim [25] found that smaller pore sizes resulted in a smaller distance between the cell walls; thus, the resistance to deformation under the external force increased with the decrease in the size of the cells. The addition of fillers to the PUR causes a reduction in cell-wall thickness, which was reflected in the strength test results of the foams [26].

The particle size of the fillers is an interesting aspect in PUR foam production. Latinowo et al., [27] investigated calcium carbonate particle size effects on PUR properties. It was found that the filler content and the particle sizes strongly affected the cell geometry structures of the foam. Javni et al. [28] reported that microfillers such as microsilica and microclay did not significantly change the cell structure or the morphology of hard domains [27,29,30,31]. In order to investigate the influence of particle size on structure, imaging techniques such as SEM (scanning electron microscope) [32,33] and X-ray computed tomography are very useful.

The use of X-ray computed tomography (CT) is an effective method for obtaining a model that represents porous microstructures [34] and dispersion levels of fillers. Mirski et al. [7] used X-ray computed tomography in order to investigate the dispersion of wood particles in the PUR matrix. This method was also used by Serban et al., [35] to illustrate the distribution of fibers in PUR foam. Furthermore, the X-ray CT also investigated how different types of carbon affect the porosity of PUR foams [36]. It is worth emphasizing that the X-ray CT has been previously performed to observe the microstructures of open-cell polyurethane foams [37]. Moreover, CT was also used for other porous materials [38,39,40].

This paper is a continuation of the previous research conducted by the authors concerning the possibility of using wood particles with different amounts as a filler in PUR foams [7].

The aim of this study was to apply the waste wood particles (WP) from primary wood processing characterized by different sizes as a filler for open-cell PUR foams. The effects of wood particle introduction on the kinetics of the foaming process and apparent density and cellular morphology of PUR foams were examined and discussed.

## 2. Materials and Methods

### 2.1. Materials

A two-component foam system PUREX-WG 2017 (Polychem System, Poznań, Poland) was used in the research. One of the components was a polyol (component A). The isocyanate component (component B) was polymeric methylenediphenyl-4,4′-diisocyanate, consisting of 31.14% free isocyanate groups.

**Figure 1:**
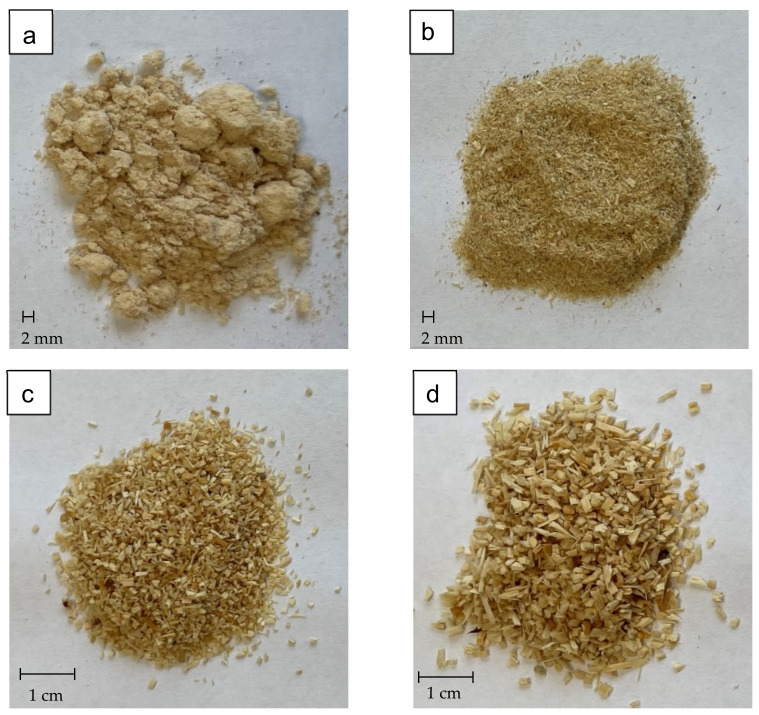
Wood particles used as a filler: (**a**) 0.05–<0.125 mm; (**b**) 0.125–<0.315 mm; (**c**) 0.315–1.25 mm; (**d**) >1.25–2.0 mm.

As a filler, Scots pine wood particles of various sizes were used (Table 1, Figure 1). Their moisture content (MC) of WP was at a range 0.2–0.5%. The wood particles were added to the foam at the concentrations of 10% (weight ratio). The amount of filler was determined based on previous studies conducted by the authors [7]. The reference variant was the foam made of A and B components, without the addition of any filler.

### 2.2. Preparation of PUR Composite Foams

The PUR foams were produced in accordance with the manufacturer’s recommendations, i.e., A:B = 100:100 with a 10% addition of wood particles. For this purpose, the reaction mixture was prepared by mixing 10% wood particles with component B (first stage) and after that component A was added and mixed (second stage). The reaction mixture was stirred with a low-speed mechanical stirrer at 1200 rpm for 10 s (for each stage), at a temperature of 23 °C. Then, it was transferred into a form with internal dimensions of 250 × 250 × 130 mm^3^. Each variant of foam was prepared in four replicates. The obtained foams were cut into specimens of the dimensions necessary for testing their properties with a band saw (Holzstar, Hallstadt, Germany).

### 2.3. Kinetics of PUR Foaming

The influence of wood particle size on the foaming process of PUR foams was evaluated. For this purpose, parameters such as start of growth, gel time, time of growth, tack-free time, and foaming temperature were determined. The definitions of the parameters are explained below:

- Start of growth: the time it takes for the mixture to start to increase;
- Cream time: the time after mixing the components when the mixture becomes a cream color;
- Gel time: the time in which, after touching with a glass rod, it is possible to remove the so-called “polyurethane thread”;
- Time of growth: time after the maximum foam growth is achieved;
- Tack-free time: the time after the foam solidifies completely;
- Foaming temperature: the temperature measured when the foam grows.

Each parameter was measured in triplicate and then the mean value was calculated.

### 2.4. Characterization of PUR-Wood Particle Composite Foams

The apparent density of PUR composite was determined according to EN ISO 845 [41] standards. For this purpose, samples with dimensions 50 × 50 × 50 mm^3^ were prepared and measured with a thickness gauge (accuracy ± 0.01 mm) and weighted on analytical balance (accuracy ± 0.001 g).

The cellular structure was determined by using scanning electron microscope (SEM) and X-ray tomography techniques. SEM analyses were conducted with the use of an SU3500 Hitachi microscope (Hitachi, Tokyo, Japan). The cell sizes of the PUR composite were determined using Motic Images Plus 3.0 software (Motic, Hongkong, China).

In order to evaluate the dispersion of wood particles in the PUR composites, the X-ray tomography method was used. For this purpose, samples with dimensions 50 × 50 × 50 mm^3^ were scanned with the use of a Hyperion X9Pro tomography (MyRay, Italy). The resolution and lamp voltage were 0.3 mm and 90 kV, respectively.

## 3. Results and discussion

### 3.1. Density

**Figure 2:**
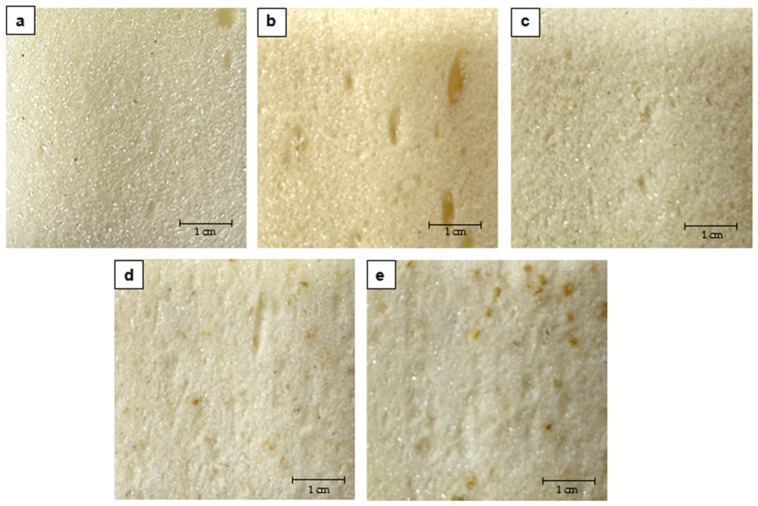
PUR-WP composite structures with different sizes of wood particle: (**a**) reference sample; (**b**) 0.05–<0.125 mm; (**c**) 0.125–<0.315 mm; (**d**) 0.315–1.25 mm; (**e**) >1.25–2.0 mm.

It can be noticed that the produced foams were characterized by a relatively well-formed structure. The foam with the addition of the finest wood particle (fraction 0.05–<0.125 mm) was characterized by the structure most similar to the pure PUR foam (Figure 2). It resulted from the fact that there was no difficulty in preparing a homogeneous mixture of foam components and a finer fraction of wood particles.

As expected, the introduction of wood dust into the foam structure also changed its density. It has been shown that, despite the use of the same amount of wood particles (10 wt.%) but differing in size, the density of foam gradually increases (Table 2). It follows that larger dust particles to a greater extent limit the expansion of the foam cells and thus the entire structure of the foam, which consequently loads the foam structure and increases its apparent density. In the case of PUR foams with fractions >1.25–2.0 mm, the increase in density was as much as 56% in relation to pure foam.

### 3.2. Kinetics of PUR Foaming

It was found that the introduction of a wood filler into the polyurethane matrix (regardless of its size) led to elongation most of the time, characterizing the foaming process (Table 3). It is especially visible in the case of the smallest fraction (variant 1). The gel time, the growth time, and the tack-free time were significantly longer by 67%, 73%, and 62%, respectively, in comparison with the reference sample. On the other hand, increasing the size of the filler particles helps to shorten the times mentioned above, while, in the case of the 0.315–1.25 mm and >1.25–2.0 mm fractions (variant 3 and 4), these parameters are similar. The addition of the finest dust fraction reduced the foaming temperature by 12 °C but did not limit its growth (Table 3). From the chemical point of view, wood is characterized by the presence of functional groups that are able to react with isocyanate groups. It can affect the proper stoichiometry of the reaction and limit the release of blowing agents (CO_2_) [19,42,43]. As a result, the reduction in foam expansion as the size of WP particles increases was observed. Therefore, taking into account the times and temperatures characteristic for the PUR foaming process, it can be concluded that the addition of the various dust fractions 0.05–<0.125 mm to the foam slows down the exothermic foaming reaction. It was observed for fractions 0.05–<0.125, 0.315–1.25, and >1.25–2.0 it resulted from the reduction of the amount of heat released during the reactions occurring in the latent stage, i.e., in the time counted from the moment of mixing the foam components to the start of its growth [44].

### 3.3. Morphology of the PUR-WP

**Figure 3:**
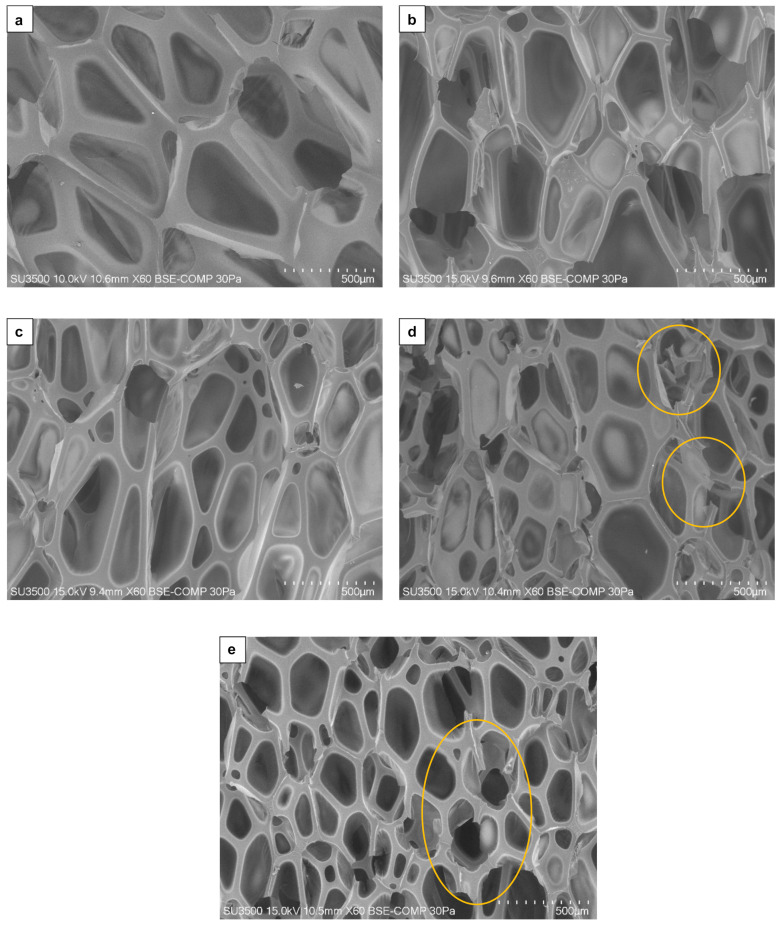
SEM images of PUR-WP composite structure: (**a**) reference sample; (**b**) 0.05–<0.125 mm; (**c**) 0.125–<0.315 mm; (**d**) 0.315–1.25 mm; (**e**) >1.25–2.0 mm. Cracks are marked with yellow circles.

The research shows that the morphology of the PUR-WP foam, in addition to the amount of wood filler introduced [7], is also influenced by its particle size. Figure 3 presents SEM photography of PUR-WP composites. The presented photos clearly show changes in the size of the cells. With the addition of larger sized particles of wood, the cell sizes decreased.

**Figure 4:**
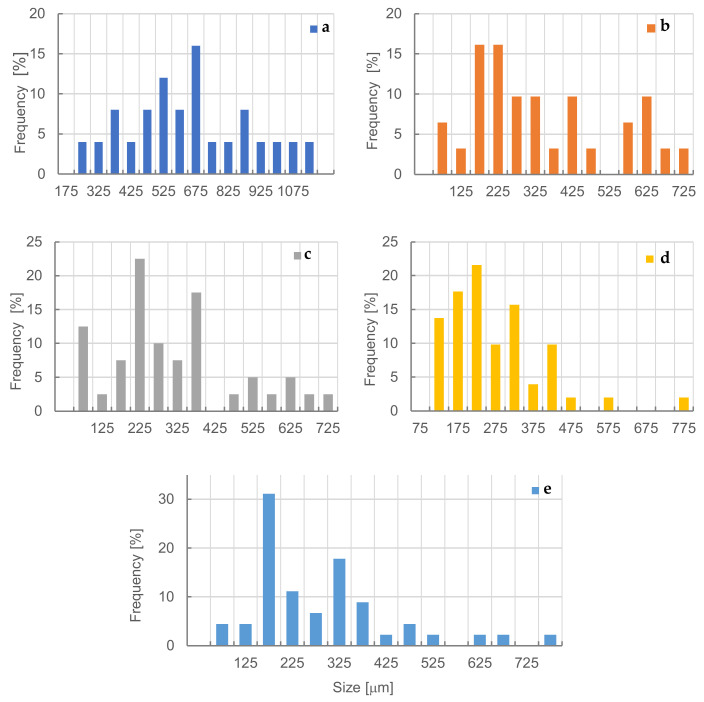
Frequency of occurrence of different cell sizes in PUR-WP composites. (**a**) reference sample; (**b**) 0.05–<0.125 mm; (**c**) 0.125–<0.315 mm; (**d**) 0.315–1.25 mm; (**e**) >1.25–2.0 mm.

As can be seen (Figure 4) in the structure of the reference PUR foam, the largest number of cells was recorded in the range of their average size between 500 and 700 µm, with the maximum frequency within the range of 650–700 µm. Similarly, to previous studies [7], the addition of dust causes the formation of cells of much smaller sizes. Kairyte et al. [2] also observed that the addition of fillers caused the reduction in the size of the average cell. In general, it can be stated that the use of filler reduced the cell cross-sectional area. This reduction is most visible in the case of fractions 3 and 4, for which the cell sizes were significantly smaller than in the reference sample. The size of cell was 225 µm and 175 µm for variant 3 and 4, respectively. In the case of foams with the addition of dust of 0.315–1.25 mm and >1.25–2.0 mm fractions, the appearance of defective cells with damage to the cell walls and struts (visible cracks) is also noticeable (Figure 3d,e). Członka et al. [45] reported that cracks could be caused by the agglomeration of the fillers. A similar effect was also observed in the research on rigid foams [26].

**Figure 5:**
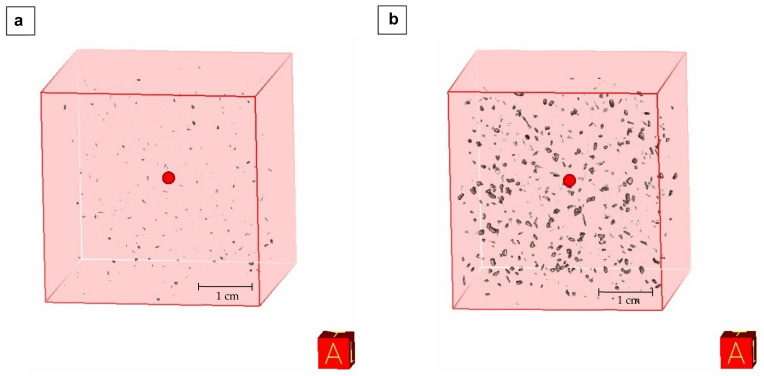
Computer tomography images of filler distribution and agglomeration in PUR-WP composite: (**a**) 0.315–1.25 mm; (**b**) >1.25–2.0 mm. Red box with letter A shows the sample orientation.

During the analyses of the foam structure, it is worth paying attention to the distribution of the filler in the foams. X-ray tomography imaging allows for a non-destructive analysis of polymeric foams and provides fully three dimensional imaging of the structure. The distribution of 0.315–1.25 mm and >1.25–2.0 mm particles in foams is presented in Figure 5. The difference in the wood particle size is noticeable, and images obtained by using a computed tomography clearly demonstrate that the WP are well-dispersed within the foams’ structures. As can be seen from the computed tomography images, this distribution could contribute to the appearance of cracks in the cells and consequently to the formation of cells with smaller diameters (Figure 3); wood particles weaken the cell structure and lead to the formation of cracks. SEM images show an increase in the number of open cells and damaged cells with damaged walls. Moreover, the use of larger size filler particles also causes cell breakage due to incomplete incorporation into the PUR matrix [25,46]. The regular shape of the particle used in the research contributed to a high degree of dispersion of its particles in the PUR-WP matrix [7].

A very important aspect is the fact that the produced foams were characterized by the ordered structure. Within one fraction, neither disproportions nor unevenness in the distribution of wood particles were observed.

## 4. Conclusions

This article focused on the morphological features of PUR-wood particle composite foams. Wood particles with different sizes were used as a filler.

It was found that the addition of WP in the amount of 10% led to an increase in density; it increased as the size of particles increased. The highest density was observed for the >1.25–2.0 mm fraction. It resulted from the fact that larger dust particles to a greater extent limited the expansion of the foam cells, which consequently loaded the foam structure and increased its density.

The research showed that the morphology of the PUR-WP foam is strongly connected with wood particle size used as a filler. The difference in the number and size of cells in PUR-WP composites also depended on the wood particle size. It was observed that, in the case of fractions 3 and 4, the highest frequency of the largest proportion of cells with smaller dimensions was observed than in the reference sample. The sizes of cells were 675 µm, 225 µm, and 175 µm for the reference sample, variant 3, and variant 4, respectively.

X-ray computer tomography clearly demonstrates that the WP are well dispersed within the foams’ structures.

## Figures and Tables

**Table 1:** Wood particle size used as a filler.

Variant	Particle Size [mm]
0	no WP added
1	0.05–<0.125
2	0.125–<0.315
3	0.315–1.25
4	>1.25–2.0

**Table 2:** Density of manufactured PUR-WP composites.

Variant	Density [kg/m^3^]
0	21.6 ± 0.5
1	27.1 ± 1.3
2	30.1 ± 0.8
3	29.1 ± 0.1
4	33.7 ± 1.0

**Table 3:** The time characterizing the foaming process.

Parameter	Filler fraction				
	0	1	2	3	4
Cream time (s)	4.0 ± 0.0	3.0 ± 0,0	2.3 ± 0.6	3.3 ± 0.6	3.3 ± 0.6
Growth start (s)	41.7 ± 2.1	38.0 ± 1.7	23.7 ± 3.1	26.3 ± 2.9	30.7 ± 2.1
Gel time (s)	103.3 ± 2.5	172.7 ± 4.6	142.7 ± 8.1	129.0 ± 9.5	125.7 ± 6.7
Time of growth (s)	140.7 ± 7.5	243.0 ± 7.0	180.0 ± 0.0	173.7 ± 3.1	170.5 ± 7.8
Tack-free time (s)	165.3 ±10.1	268.7 ± 5.7	256.0 ± 13.5	190.0 ± 10.6	194.3 ± 9.5
Temperature of foaming (°C)	105.5 ± 0.7	93.0 ± 4.2	105.5 ± 4.9	99.0 ± 5.7	90.5 ± 0.75
Growth (mm)	150.0 ± 6.0	150 ± 5.0	150 ± 2.0	145.0 ± 4.0	134.0 ± 5.0

## Data Availability

Not applicable.
